# The effect of garden use on quality of life and behavioral and psychological symptoms of dementia in people living with dementia in nursing homes: a systematic review

**DOI:** 10.3389/fpsyt.2023.1044271

**Published:** 2023-04-12

**Authors:** Melanie van der Velde-van Buuringen, Rosalie Hendriks-van der Sar, Hilde Verbeek, Wilco P. Achterberg, Monique A. A. Caljouw

**Affiliations:** ^1^University Network for the Care Sector Zuid-Holland, Leiden University Medical Center, Leiden, Netherlands; ^2^Department of Public Health and Primary Care, Leiden University Medical Center, Leiden, Netherlands; ^3^Zorginstellingen Pieter van Foreest, Delft, Netherlands; ^4^Department of Health Services Research, Care and Public Health Research Institute (CAPHRI), Faculty of Health, Medicine and Life Sciences, Maastricht University, Maastricht, Netherlands; ^5^Living Lab in Ageing and Long-Term Care, Maastricht University, Maastricht, Netherlands

**Keywords:** dementia, nursing homes, quality of life, behavioral and psychological symptoms of dementia, garden use, systematic review

## Abstract

**Objectives:**

Considering the importance of going outside in a natural environment for people in general, and people living with dementia in particular, we want to unravel the aspects by which garden use affects quality of life (QoL) and behavioral and psychological symptoms of dementia (BPSD) in people living with dementia in nursing homes.

**Design:**

Systematic review.

**Setting and participants:**

People living with dementia in nursing homes.

**Methods:**

This systematic review followed the Preferred Reporting Items for Systematic Reviews and Meta-Analyses (PRISMA) guidelines. Eight electronic bibliographic databases were searched (May 2022). Quantitative, qualitative and mixed-methods studies describing the effect of garden use on QoL, BPSD, or other outcomes related to QoL or BPSD in people living with dementia in nursing homes were included. The methodological quality of individual studies was assessed with the Mixed Methods Appraisal Tool (MMAT) and a narrative synthesis of the results was performed.

**Results:**

After screening title and abstract (*N* = 498), and full-text assessment (*N* = 67), 19 publications were included. These described 17 studies and three types of interventions: (1) interventions regarding the evaluation of effects of specifically designed nursing home gardens, (2) participation of the people living with dementia in outside activities, and (3) other interventions, for example, garden visits and different seasons.

**Conclusions and implications:**

Overall, first studies appear to suggest positive effects of garden use on QoL, BPSD, or other outcomes related to QoL or BPSD (stress, sleep, and mood) in people living with dementia in nursing homes. However, consensus regarding measurements and key outcomes, taking into account the physical, social, and organizational aspects when designing the garden use intervention, is necessary for the reliable evaluation of these interventions.

**Systematic review registration:**

https://www.crd.york.ac.uk/prospero/display_record.php?RecordID=283267, identifier: CRD42021283267.

## 1. Introduction

Dementia is categorized as a major neurocognitive disorder, and is an irreversible disorder with a progressive decline in various cognitive functions that influences intellectual, social and physical functioning (1, 2). Most of the people living with dementia in nursing homes spend their day inactive in a lying down or sitting position, and on average more than 90% of the residents stay inside their ward during the day (3). They experience a major loss of quality of life (QoL), defined by “the multidimensional evaluation of the person-environment system of the individual, in terms of adaptation to the perceived consequences of the dementia” (4, 5). Some of the aspects that influence QoL are behavioral and psychological symptoms of dementia (BPSD), for example agitation (2, 6). BPSD is defined as “signs and symptoms of disturbed perception, thought content, mood, and behavior” (6, 7). Possible causes of the symptoms are neurobiologically related disease factors, unmet needs such as hunger or pain, caregiver factors and environmental triggers (7).

Various interventions have been developed to tackle the problems as mentioned above. One of these interventions is the passive and active use of gardens of nursing homes (8). Garden use consists of a variety of activities, some individual, some communal. The definition of gardens and garden use differs widely in terms of scale, function, and activity (9). Gardens are often thought of as intimate private spaces attached to private households but they can also be large private or formal gardens part of nursing homes (9). There are different possibilities regarding the use of gardens in nursing homes, for example horticultural therapy, which uses plant-related activities as a therapeutic modality to achieve goals (10), or green care farms that combine agricultural with care activities (11).

In this systematic review the term garden use refers to any activity in the nursing home garden that is a person-centered activity and fits within the usual activities in daily nursing home practice, meaning going outside into the nursing home garden and doing an activity outside that is usually done inside. Examples of person-centered garden activities are sitting, walking, having a conversation, drinking a beverage, having lunch, gardening, or receiving therapy (12). Person-centered care is a care approach built around the needs of an individual. It recognizes that all people are unique and have their own personal needs. The task of the caregivers is to be aware of behaviors that undermine the person's wellbeing (and to do that as little as possible) and enhance the person's wellbeing (and to do that as much as possible) to deliver optimum levels of care. The activities are tailored to the residents' wishes and preferences (13, 14).

Being in the garden can provide a physical and psychological distance from stress and attention evoking stimuli (15). There are different theories about how being in a natural environment such as a garden can promote more rapid and complete restoration of (the consequences of) stress than other environments, but two contrasting theories dominate this field (15). The psychoevolutionary theory places emphasis on stress reduction whereby contact with nature can very rapidly evoke positive affect, which in turn blocks negative thoughts and feelings and fosters reduction of physiological activation (15–17). The attention restoration theory places emphasis on recovery of the capacity to focus attention, whereby effortless attention engaged by intrinsically interesting aspects of nature enables rest for a fatigued neurocognitive inhibitory mechanism engaged when wilfully directing attention (15, 18).

In recent years, interest in the effects of garden use on people living with dementia in nursing homes has increased. More and more studies from different disciplines are finding positive effects of different aspects of garden use. For example, one review suggested an overall positive effect of the creation of dementia-friendly gardens on agitation, apathy and engagement, despite concerns about the methodological approaches (19). Another review examined the barriers and facilitators affecting nursing home residents' use of outdoor space, as perceived by residents, their family members, and staff (20). This study showed that, in addition to specific aspects in the design of the garden, cultural change at an organizational level is also necessary, for example by addressing perceptions of safety. Whear et al. (21) showed promising results for the effect of garden use on agitation.

Despite the increasing number of studies on this topic, there is no systematic review done of recent specific data on the effect of garden use on QoL and BPSD in people living with dementia in nursing homes. Considering the importance of going outside in a natural environment for people in general (15), and people living with dementia in particular (21), we want to unravel which aspects of garden use affect QoL and BPSD in people living with dementia in nursing homes. This systematic review therefore addressed the following research question: “What is the effect of garden use on QoL and BPSD in people living with dementia in nursing homes?”

## 2. Methods

This systematic review was conducted and reported following the Preferred Reporting Items for Systematic Reviews and Meta-Analyses (PRISMA) guidelines (22). The search and analysis methods were specified in advance in a protocol. The protocol is registered in the International Prospective Register of Systematic Reviews (PROSPERO; CRD42021283267).

### 2.1. Search strategy

The search strategy was developed together with an information specialist and included terms related to garden use, dementia, QoL, BPSD, and nursing homes. For the complete search strategy see the Supplementary material online attached to the electronic version of this paper. Searches were conducted in eight electronic bibliographic databases for the period 1946 to May 2022: PubMed, MEDLINE, Embase, Web of Science, COCHRANE Library, Emcare, PsycINFO, and Academic Search Premier. The search in the electronic bibliographic databases was conducted on May 12, 2022.

### 2.2. Eligibility criteria

Research articles describing the effect or measuring the effect of the intervention of garden use (*outdoor spaces, outside, wander garden, therapeutic garden, and healing garden in the nursing home environment*) on QoL (*wellbeing and life quality*), and BPSD (*BPSD, neuropsychiatric symptoms of dementia*) in people living with dementia in nursing homes [*nursing homes by the definition of Sanford et al*. (23) “*A facility with a domestic-styled environment that provides 24-h functional support and care for* people *who require assistance with ADLs and who often have complex health needs and increased vulnerability,” institutional care*] were eligible for inclusion. In addition, quantitative, qualitative and mixed-methods studies in English/Dutch/German/French were eligible for inclusion. Letters to the editor, reviews, studies describing the effects of horticultural therapy, or taking place at facilities without 24-h functional care were excluded.

### 2.3. Study selection

Two researchers (MVB and RHS) independently assessed which studies retrieved in the searches met the inclusion criteria based on titles and abstracts. This was followed by full-text assessments. Differences were discussed until consensus was reached, and when necessary by consulting a third researcher (MAAC).

### 2.4. Methodological quality of individual studies

The Mixed Methods Appraisal Tool (MMAT) version 2018 (24) was used to assess the methodological quality of all included individual studies. One researcher (MVB) carried out the assessment, which was checked by a second researcher (RHS). Again, consensus was reached through discussion, and when necessary by consulting a third researcher (MAAC).

### 2.5. Data extraction and analysis

A standardized data extraction form was developed to extract the data of the included studies. A description of the included studies was summarized in a table by extraction of year and country of publication, study design, study population, intervention, outcome measures, and study quality (MMAT). Studies were not excluded from the review based on their quality.

Included studies were anticipated to be very diverse in terms of intervention and outcome measures, making pooling impossible. Therefore, a narrative synthesis of the findings was given in a table structured by outcome (study design, participants, type of intervention, QoL, BPSD, other outcomes related to QoL or BPSD, and methodological quality) and description of the aim/objective of the study and main findings. This synthesis was carried out by two researchers (MVB and RHS), and a third author (MAAC) was available if agreement was not reached.

## 3. Results

### 3.1. Study selection

**Figure 1:**
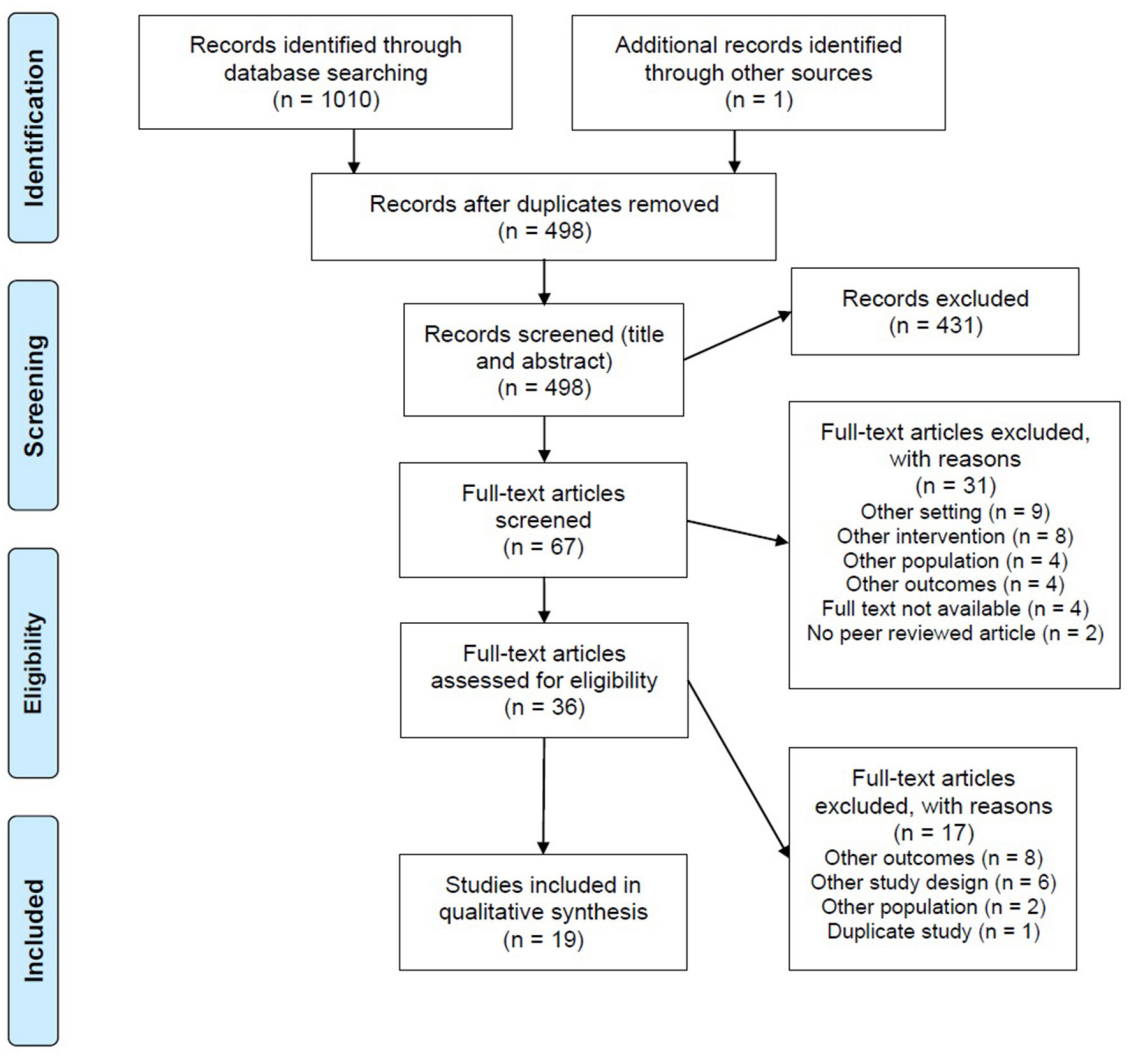
Flow diagram of the study screening and selection.

The process of screening and selection is shown in the flow diagram in Figure 1. After removal of duplicates, 498 publications remained. After screening title and abstract on inclusion criteria, 431 publications were excluded. The remaining 67 publications were screened full text, after which 31 publications were excluded. The remaining 36 publications were assessed for eligibility and finally 19 publications were included in this review.

### 3.2. Study characteristics

The 19 publications included in this review described 17 different studies. The publications of Detweiler et al. (25), Detweiler et al. (26), and Murphy et al. (27) reported on different aspects of the results of the same study. The characteristics of the included studies are presented in Table 1.

**Table 1:** Description of included publications (*N* = 19).

**References**		**Methods**		**Outcome measures**			**Quality**
**Publication (country)**	**Study design**	**Participants**	**Intervention**	**QoL**	**BPSD**	**Other**	**MMAT score (%)**
Bourdon and Belmin (28) (FR)	Non-randomized cluster-controlled trial (Pilot study)	*n* = 120 PWD^*^ (39 PWD control group; 41 PWD conventional sensory garden group; 40 PWD enriched garden group; no significant differences in characteristics between groups)	- PWD were assigned to one of three groups based on the location of their room and its proximity to one of the two gardens: (1) close to conventional sensory garden, (2) close to enriched garden, (3) neither.- Staff encouraged PWD assigned to the conventional sensory garden group and the enriched garden group to visit their respective gardens at least 4 times a week. Usual care for control group.- 6 Months during spring and summer.	-	-	- Independence for activities of daily living: ADL^‡^- Risk of falls: Unipodal stance and TUG^§^- All measurements rated by two observers (a psychologist and an occupational therapist who were independent of the research team)	80
Calkins et al. (29) (USA)	Before-and-after study; Non-randomized study	*n* = 17 PWD (15 females, two males; MMSE *M* = 10.5) *n* = 32 staff members (30 females, two males)	- Four conditions: A) winter/no activityB) winter/inside activityC) summer/no activityD) summer/outside activity - Activity minimum of 30 min- Data collection A and C 1 week, B and D 2 weeks.	-	CMAI (rated by day and evening shift staff members, research assistant)^||^	- Sleep: Actilume-L devices (movement and light); PSQ (rated by night shift staff members)^^**^^- Mood: Facial Affect Rating Scale (rated by research assistant)	100
Cohen-Mansfield (30) (USA)	Non-comparative study; Quantitative descriptive study	*n* = 320 staff members of facilities (66% directors, 13% administrators, 16% other position, 6% not specified)	National survey in long-term care facilities with outdoor areas investigating the characteristics and features of these areas and how they relate to their perceived impact on their users.	-	-	Experience with outdoor area: Survey (with staff members)	80
Connell et al. (31) (USA)	Quantitative randomized controlled trial (Pilot study)	*n* = 20 PWD [one female, 19 males; Age range 64–90; Outdoor MMSE *M* = 11.7; Indoor MMSE *M*: 18.9 (Difference *p* = 0.05)]	- Two groups: Outdoor activity program in existing outdoor space vs. indoor activity program in existing activity space.- Activity same for both groups, namely with horticultural focus.- Duration of 10 days, with 1 h activity.- Data collected at baseline (10 days) and during intervention (10 days)	-	CMAI (rated by primary care staff members who were interviewed by research team)	Sleep: Wrist actigraphs with photocells	60
Cox et al. (32) (AU)	Mixed- methods study	Quantitative part: *n* = 24 PWD (23 females, one male; 29% Resolution Stage 3; 38% Stage 2; 33% Stage 1) Qualitative part: *n* = 12 staff members and relatives	Quantitative part: - Each participant experienced each of the three activities (living room, garden, Snoezelen room) accompanied by a caregiver during 3 individual 16-min sessions, thus with total of nine sessions per resident.- One session a day between 10:00 and 15:00 h Qualitative part: Both groups were asked the same set of questions (own responses to environment, their impressions of residents' responses to environment, aspects of environments which were enjoyed by themselves or the residents)	-	-	Quantitative part: - ARS (rated by two trained observers)^††^ Qualitative part: - Interview with open -ended questions (with staff members and relatives)	20
Detweiler et al. (25) (USA) used data from Detweiler et al. (26) (USA)	Before-and-after study; Non-randomized study	*n* = 28 PWD (28 males; Age *M* = 80.5; Ambulation category = 17 ambulatory, two merry walker, nine wheelchair)	- Baseline year: 1 year of observations at the closed dementia unit without a wander garden.- Observation year: 1 year of observations at the closed dementia unit (after adding a wander garden) with a wander garden.	-	Number of falls: Fall severity score based on the Institutional Fall Committee ratings (obtained by researcher)- Scheduled psychiatric medications (obtained by researcher)	-	80
Detweiler et al. (26) (USA)	Before-and-after study; Non-randomized study	Baseline year: *n* = 34 PWD (34 males; Age *M* = 80.71; Ambulation category = 21 ambulatory, two merry walker, 11 wheelchair) Observation year: *n* = 29 PWD (due to mortality) *n* = 42 staff members and relatives	- Baseline year: 1 year observations at the closed dementia unit without a wander garden.- Observation year: 1 year observations at the closed dementia unit with a wander garden.	-	- CMAI (rated by the same team member, who saw all patients every day for multiple hours of activity)- Incident reports (level 1 least serious to 4 most serious; obtained by researcher)-PRN^‡‡^ medications (obtained by researcher)- Survey (with staff and family)	-	80
**Publication (country)**	**Study design**	**Participants**	**Intervention**	**QoL**	**BPSD**	**Other**	**MMAT score (%)**
Dyer et al. (33) (AU)	Non-comparative study; Quantitative descriptive study	*n* = 541 residents (348 PWD (64.3%); 453 PWD or PAS-Cog^§§^≥ 5 (83.7%); 403 Females (74.5%); Age *M* = 85.5)	Survey at nursing homes with and without independent access to outdoor spaces.	HR-QoL^||||^ assessed with EQ-5D-5L (rated by the residents where possible, or a proxy family member where necessary)^***^	-	-	100
Edwards et al. (34) (AU)	Mixed- methods study	*n* = 10 PWD (nine females, one male; 4 PW severe D; 3 PW moderate D; 3 PW mild D; Age range = 79–90)	- Evaluation of effects of a therapeutic garden designed specifically for PWD on the basis of results of literature review to increase QoL.- Measurements 3 months prior to new garden and 3 months post new garden construction.- Pre: Log sheets and observations over a 12-day period in autumn with frequency use old garden by residents, staff and visitors.- Post: Log sheets and observations over a 12-day period in following spring with frequency use new garden by residents, staff and visitors, in same weather conditions.	DEMQOL (Proxy; If the resident was assessed according to the MMSE as having mild dementia they completed it themselves with a trained staff member assisting, otherwise a family member assessed it with the assistance of a staff member trained in the administration). ^†*††*^	CMAI (rated by trained staff members)	- Depression: SCDD (rated by trained staff members)^‡*‡‡*^- Open-ended questions interview (with staff members and family)	20
Evans et al. (35) (UK)	Mixed- methods study	Online survey: *n* = 144 survey responses (average of 40.6% PWD: 50% extra care housing; 25% residential care homes; 13% nursing care homes; 3% retirement villages; 1% continuing care schemes; 8% unknown) Interviews: *n* = 19 residents (seven extra care housing; 12 care home) *n* = 16 staff members (seven extra care housing; nine care home)	- Following a review of literature, an online survey was developed (demographics; current green dementia care experiences and activities; barriers and enablers to providing green dementia care; perceived impacts of green dementia care).- In depth-case study research was carried out at three care homes and three extra care housing schemes (examples of good practice). Interviews were conducted.	- Online survey: “Perceived impacts of green dementia care” (with staff members)	-	- Online survey: “Perceived impacts of green dementia care” (with staff members)- Interviews (with residents and staff members)	20
Goto et al. (36) (JP)	Before-and-after study; Non-randomized study	*n* = 32 PWD (16 hospital and 16 nursing home; 28 females, four males; 8 PW severe D; 14 PW moderate D; 8 PW mild D; 2 Unknown; Care-need level of 1–5 according to standard of Ministry of Health of Japan; Age *M* = 91; MMSE *M* = 12; No significant difference in average age, MMSE score, lifestyle and education between the two sites)	- Construction of two Japanese gardens; Hospital garden and Terrace (nursing home) garden.-Test 1 (T1) was conducted in 2 weeks prior to construction of garden (April).-Test 2 (T2) was conducted in 2 weeks post construction of garden (June).-Test 3 (T3) was identical to T2, except that the subjects faced the gardens with the glass doors closed (October).-For all tests, subjects were escorted to view the garden together with caregiver and researcher for 15 min, two times per week at approximately the same time during daytime hours.	-	-	- Physiological stress: Heart rate- Behavior: Behavioral Assessment Check List (rated by researcher and primary care staff member)	80
Hendriks et al. (37) (NL)	Qualitative study	Focus groups: - *n* = 34 PWD (25 females, nine males; Age *M* = 81.22; 10 Nursing Home, 11 meeting center, 13 daycare center) Pilot study decision tool: *n* = 13 PWD with one or more behavior or mood problems [based on NPI-Q^§§§^; seven females, six males; Age *M* = 78.09; 4 PWD from Nursing home (5 meeting center, 4 day care)]	Focus group study - An executed review was input for a discussion guide regarding experiences and activities in nature that was applied in the focus group study.- Focus group lasted ~1 h - Pilot study of a decision tool for personalized nature activities - Based on the outcomes of the decision tool PWD were assigned to 1 of 3 designed example activities (nature walk, gardening, and sensory activation in nature).- All activities were personalized- All activities were in a group context.- PWD from NH only participated in the nature walk (day care gardening; meeting center gardening and nature walks)- Executed during spring	- Themes of focus group interviews were categorized into QoL domains [(38); with residents]- Semi-structured interviews focusing on their experience of and satisfaction with the activity (with residents)	-	Mood: OERS^||||||^ and the Interact instrument (rated by the researchers)	100
Hernandez (39) (USA)	Mixed- methods study	Interviews: *n* = 40 staff members and relatives Observations: *n* = 59–79 PWD	- Effects of “therapeutic garden” concept- Post occupancy evaluation after installation of two gardens: Garden View and Sunshine Center - Interviews with staff and families of 20–45 min about behavior - Observations during common as well as uncommon hours from 6 to 8-h blocks of time until saturation was achieved.	- Behavior: Interviews (with staff members and relatives)	-	- Behavior: Interviews (with staff members and relatives)- Emotional reactions: AARS (rated by the researcher)^****^	0
Liao et al. (40) (USA)	Mixed- methods study (Pilot study)	*n* = 42 staff members (42 females; 35 free garden use group; seven unfree garden use group)	Pilot study evaluating the effects of garden visits on outcome measures *via* semi- structured questionnaires.	-	Semi-structured questionnaire (with staff members)	Semi-structured questionnaire (with staff members)	20
Mather et al. (41) (CA)	Before-and-after study; Non-randomized study	*n* = 10 PWD (seven females, three males; Age *M* = 83)	- Pre-post comparison of garden access across the summer period. In the winter access was limited due to cold and snow.-Garden was constructed a year prior to start of study.- Measurements were done for periods of 1 week pre-, mid-, and post-summer.	-	Baumgarten, Becker and Gauthier's checklist, modified (rated by trained staff members)	Baumgarten, Becker and Gauthier's checklist, modified (rated by trained staff members)	50
Motealleh et al. (42) (AU)	Mixed- methods study (Case study)	Quantitative part: *n* = 10 PWD (nine females, one male; Age *M* = 81.7; 2 mild impairment (PAS score); 6 moderate; 2 severe) Qualitative part: *n* = 10 PWD (nine females, one male; Age *M* = 81.7; 2 mild impairment (PAS score); 6 moderate; 2 severe) *n* = 10 staff members	Quantitative part: - Improving garden based on dementia-friendly environment (DFE) characteristics- Researcher accompanied each participant from their bedroom into the improved garden-4-week intervention with daily (Monday-Friday) 60-min session Qualitative part: - Post-intervention individual semi-structured interviews with PWD and staff members to understand perceptions of the improved garden; and with staff members to determine their views on the effect of the improved garden on PWD	-	CMAI-SF (rated by primary care staff members)- PEAR (rated by the researcher)^†*†††*^	- Engagement: EPWDS (rated by the researcher)^‡*‡‡‡*^	100
Murphy et al. (27) (USA) used data from Detweiler et al. (26) (USA)	Before-and-after study; Non-randomized study	Baseline year: *n* = 34 PWD (34 males; Age *M* = 80.71; Ambulation category = 21 ambulatory, two merry walker, 11 wheelchair) Observation year: *n* = 29 PWD (due to mortality)	- Baseline year: 1 year observations at the closed dementia unit without a wander garden.- Observation year: 1 year observations at the closed dementia unit with a wander garden.	-	CMAI (rated by the same team member, who saw all patients every day for multiple hours of activity)	-	80
van der Velde-van Buuringen et al. (12) (NL)	Mixed- methods study (Feasibility study)	Quantitative part: *n* = 20 PWD (13 females, seven males; Age *M* = 85.2; GDS^§§§§^ 5–7) Qualitative part: Caregivers, psychologist, elderly care physician, occupational therapist, physiotherapist, registered nurse, managers.	- Garden-use intervention consisting of going outside for at least 30 min in the nursing home garden, for any person-centered activity that fits within the usual activities in daily nursing home practice.- The study lasted 8 weeks; the first 2 weeks were the baseline period (no instructions), between weeks 3 and 4 the intervention was implemented (researcher helped the caregivers, who were the primary coordinators of the intervention, to start planning the execution of the intervention), weeks 5 and 6 were the intervention period and final measurements were carried out at the end of week eight. During the follow-up period (weeks 7–8) the wards received no instructions or suggestions.	QUALIDEM (rated by primary care staff members, assisted by the researcher)	NPI-NH (rated by primary care staff members, assisted by the researcher)	Interviews and questionnaires about process of and experience with intervention (with staff members)	20
White et al. (43) (UK)	Before-and-after study; Non-randomized study	*n* = 28 PWD (Mid- to late-stage dementia)	- Carer-mediated exposure to a nature-rich garden (following previously established guidelines on design).	-	-	Mood: Carer-assessed score on a scale of 1–3, representing poor, medium and good, respectively (rated by primary care staff members)	60

* People living With Dementia.

‡ Activities of Daily Living.

§ Timed Up and Go.

|| Cohen-Mansfield Agitation Inventory (-Short Form).

** Pittsburgh Sleep Quality Index.

†† Affect Rating Scale.

‡‡ Pro re nata.

§§ Psychogeriatric Assessment Scale Cognitive Impairment Scale.

|||| Health-related QoL.

*** EuroQoL instrument.

†*††* Dementia Quality of Life Instrument.

‡*‡‡* Cornell Scale for Depression in Dementia.

§§§ Neuropsychiatric Inventory (-Nursing Homes).

|||||| Observed Emotion Rating Scale.

**** Apparent Affect Rating Scale.

†*†††* Person-Environment Apathy Rating Scale.

‡*‡‡‡* Engagement of a Person with Dementia Scale.

§§§§ Reisberg Global Deterioration Scale.

### 3.3. Study design

Seven publications used a before-and-after non-randomized design (25–27, 36, 41, 43), and seven had a mixed-methods design (12, 32, 34, 35, 39, 40, 42). A non-comparative quantitative descriptive study design was used in two publications (33), one used a quantitative randomized controlled trial design (31), one was a non-randomized clustered controlled trial (28), and one had a qualitative study design (37). Four studies were pilot or feasibility studies (12, 28, 31, 40).

#### 3.4. Participants

The number of participants included in the publications ranged from 10 people living with dementia (34, 41) to 541 residents [of whom 453 people living with dementia (83.7%) or a score of ≥ 5 on the Psychogeriatric Assessment Scale Cognitive Impairment Scale (PAS-Cog)] (33). Ten publications included only people living with dementia as participants (25, 27, 28, 31, 33, 34, 36, 37, 41, 43), while seven included people living with dementia as well as staff members and relatives as participants (12, 26, 29, 32, 35, 39, 42), and two only included staff members as participants (30, 40).

#### 3.5. Interventions

A wide variety of interventions are described. They can be grouped into interventions regarding the evaluation of (1) effects of specifically designed nursing home gardens, (2) participation of the people living with dementia in activities in the garden of the nursing home, and (3) other interventions. Eight studies described an evaluation of the effects of specifically designed gardens (25–28, 34, 36, 39, 42), three regarded interventions in which the people living with dementia participated in activities that took place in different environments, for example, the garden vs. the living room (29, 31, 32), and the rest of the publications described a range of other interventions, like an evaluation of the effects of garden visits (40, 43), garden visits in different seasons (29, 41), of (not) having independent access to the outdoor spaces (33), and creating and testing a decision tool for personalized nature activities (37). Furthermore (a number of), sessions were part of the intervention in only three publications (32, 36, 42).

#### 3.6. Outcome measures

Overall, there was a wide variety in outcome measures. The results of the publications were divided based on outcome: QoL, BPSD, and other outcomes related to QoL or BPSD (see Table 1). The outcome QoL was measured both quantitatively (12, 33, 34, 39), and qualitatively (35, 37, 39). The measurements of BPSD showed the most homogeneity: eight of the 10 publications used quantitative measures, of which six the Cohen-Mansfield Agitation Inventory [-Short Form; CMAI (-SF)] (26, 27, 29, 31, 34, 42). Other measurements used were the number of falls, number of incidents, scheduled and pro re nata psychiatric medications, the modified Baumgarten, Becker and Gauthier's checklist, the Person-Environment Apathy Rating Scale (PEAR), and the Neuropsychiatric Inventory Nursing Homes (NPI-NH) (12, 25, 26, 41, 42). Only three publications (also) used qualitative measures, namely a survey, a semi-structured questionnaire, and a semi-structured interview (26, 40, 42). Other quantitative QoL- or BPSD-related outcome measures used were, for example, heart rate (stress), wrist actigraphy with photocells (sleep), and the Cornell Scale for Depression in Dementia (SCDD; depression). The qualitative outcome measures consisted mostly of (semi-structured) questionnaires or surveys. Due to the considerable heterogeneity of the used measures, it is very difficult to compare the effectiveness of the different interventions.

#### 3.7. Methodological quality of individual publications

The results of the assessment of the methodological quality of individual publications are presented in Table 1. The overall MMAT score ranged from 0% (39) to 100% (29, 33, 37, 42). Of the 19 publications, one scored 0% (39), five scored 20% (12, 32, 34, 35, 40), and 13 scored 50% or higher. Most of the studies using mixed methods made insufficient use of the potential of this type of design. For example, they did not describe if and how the different components of the study were effectively integrated to answer the research question, nor did they adequately address the divergences and inconsistencies between quantitative and qualitative results. Overall, however, the independent quantitative and qualitative components of these studies were of good quality.

#### 3.8. Results of individual studies

##### 3.8.1. Quality of life

The results regarding QoL are summarized in Table 2. All six publications described a positive effect of garden use on QoL in people living with dementia in nursing homes (12, 33–35, 37, 39). Some publications show an overall positive effect of garden use on QoL (34, 39). Others found a more specific positive effect on QoL. Van der Velde-van Buuringen et al. (12) for example, found that people living with dementia showed an increase in the domain positive affect and a decrease in the domain social isolation of the QUALIDEM during the intervention period of going outside. Another example is the study by Dyer et al. (33) which found that going outdoors daily was significantly associated with better Health-related QoL (HR-QoL) of residents. However, going outdoors multiple times (1–6 times) per week but not daily was not significantly associated with better HR-QoL. Based on focus group interviews Hendriks et al. (37) found eight key themes concerning the question what kind of experiences persons living with dementia find important for their wellbeing and QoL when in nature: Pleasure, relaxation, feeling fit, enjoying the beauty of nature, feeling free, the social aspect of nature, feeling useful, and memories. In Evans et al. (35) staff members mentioned many ways in which they felt nature-based activities had a positive effect on the wellbeing of persons living with dementia, including high levels of engagement, a sense of freedom, creativity, increased social interaction, inter-generational contact with families, and the calming effect of contact with animals. Of the five publications with qualitative data (12, 33, 35, 37, 39), only two (35, 37) publications (also) asked the people living with dementia directly about their experiences, instead of only staff members and/or relatives on the behalf of the people living with dementia. Three (12, 35, 37) of the publications were suitable for further in-depth data synthesis. In these papers, five themes were identified that appear to capture the overall experiences of people living with dementia, staff members, and relatives of the effect of garden use on QoL: sense of freedom (35, 37), social interaction (35, 37), calming effect (12, 35, 37), reminiscence (12, 37), and pleasure (35, 37).

**Table 2:** Main findings on outcome measure QoL (*n* = 6).

**Outcome**	**References**	**Aim/objective**	**Main findings**
QoL	Dyer et al. (33)	“To examine the association between provision of independent access to outdoor areas at the nursing home level and actual use of outdoor areas by the residents with HR-QoL in a population of residents of Australian nursing homes with a high prevalence of dementia.”	- Going outdoors daily was significantly associated with better HR-QoL of residents. However, going outdoors multiple times (1–6 times) per week but not daily was not. Living in a NH with independent access to outdoor was also not significantly associated with better HR-QoL of residents. Simply providing independent access to outdoor areas is insufficient to achieve HR-QoL benefits for residents in nursing homes; there is a need to enable and support regular use of outdoor spaces.
	Edwards et al. (34)	“To evaluate whether a therapeutic garden can improve the quality of life of aged care residents with dementia and their carers.”	- Significant improvements in QoL of residents after creation and use of therapeutic garden. Staff, family and resident interviews elicited consistently positive feedback concerning the new garden, including observations that it had improved the QoL of the residents.
	Evans et al. (35)	“To report on a project that aimed to explore the opportunities, benefits, barriers and enablers to interaction with nature for people living with dementia in residential care and extra care housing schemes in the UK.”	- Staff also mentioned many ways in which they felt nature-based activities had a positive effect on wellbeing of PWD, namely high levels of engagement, a sense of freedom, creativity, increased social interaction, inter-generational contact with families, and the calming effect of contact with animals.
	Hendriks et al. (37)	“To develop an approach, including examples of personalizable nature activities and a decision tool to design personalized nature activities for people with dementia, and to try this out among people with behavior and mood disruptions. Which aspects of being in nature or outdoor spaces do people with dementia find important for their quality of life?”	- Concerning the question what kind of experiences PWD find important for their wellbeing and QoL when in nature, eight key themes emerged: Pleasure, relaxation, feeling fit, enjoying the beauty of nature, feeling free, the social aspect of nature, feeling useful, and memories.
	Hernandez (39)	“What effect does the garden design have on the QoL of residents living in special care units for people with dementia?”	- Value was placed on the garden (or outdoor space) as a therapeutic tool for enhancing life quality.
	van der Velde-van Buuringen et al. (12)	“To evaluate the process (usefulness, feasibility, and applicability) of going outside daily in a nursing home garden and to explore the effect of garden use on QoL and neuropsychiatric symptoms in persons with advanced dementia.”	- PWD showed an increase in the domain positive affect and a decrease in the domain social isolation of the QUALIDEM^*^ during the intervention period of going outside. No significant differences were found for the other domains of QoL. The intervention was observed to be positive for the people living with dementia in terms of improved reminiscence, less agitated behavior, a new positive habit and being more awake during the day.

* Dementia Quality of Life Instrument.

##### 3.8.2. Behavioral and psychological symptoms of dementia

The results regarding BPSD are summarized in Table 3. Seven of the 10 publications describe positive effects of garden use on BPSD (25–27, 31, 34, 40, 42). Some publications describe an overall positive effect on BPSD (26, 33), others a more specific effect, for example on the frequency of verbal agitation (31). Murphy et al. (27) showed that the degree to which the average numbers of days spent in the wander garden is associated with decreased agitation scores is dependent on baseline agitation scores and ambulation ability. There was more effect for the people living with dementia who had higher levels of agitation at the beginning of the study than for those who had lower levels of agitation. Also, when residents could walk without assistance, a low, medium and high usage of the garden reduced agitation, with a higher frequency corresponding with a greater decrease in agitation. For the merry walker chair and wheelchair users, those who had a high number of garden visits showed decreased agitation levels, but medium or low garden usage was associated with unchanged or increased agitation. Detweiler et al. (25) found variations in effects of low or high frequency garden use. The high frequency garden use group showed a decreased need for scheduled high-dose and intermediate-dose antipsychotics, eliminated and reduced the need for scheduled secondary antidepressants, and scheduled intermediate-dose hypnotics compared to the low frequency garden use group. Also, increased garden use appeared to be related to a decreased frequency and severity of falls. The rest of the publications showed no significant positive effects, or inconclusive or contradictory effects of garden use on BPSD (12, 29, 41, 42). Both van der Velde-van Buuringen et al. (12) and Motealleh et al. (42) found no significant differences in the quantitative data measured with the NPI-NH and CMAI, but when conducting semi-structured interviews with people living with dementia and staff members, reduced agitated behavior was mentioned as one of the positive results of garden use. Calkins et al. (29) found changes that were contradictory, namely fewer resident-requests for attention during the day as observed by the research assistant, but more requests for attention in the evening as observed by the evening shift staff members. An explanation given by the researcher is that because the people living with dementia are sleeping better, they don't want to go to bed as early and therefore request attention. Liao et al. (40) showed that garden visits had positive effects on behavioral problems, through multisensory stimulation, a feeling of independence, provoking a recall of memories, and relieving stress. Of the three publications with qualitative data (12, 40, 42), only one publication (42) (also) asked the people living with dementia directly about their experiences, instead of only staff members on the behalf of the people living with dementia. All three of the publications were suitable for further in-depth data synthesis, whereby one theme was identified: Garden use had a positive effect on agitation (12, 40, 42).

**Table 3:** Main findings on outcome measure BPSD (*n* = 10).

**Outcome**	**References**	**Aim/objective**	**Main findings**
BPSD	Calkins et al. (29)	“To examine the impact of increased time spent outdoors on sleep and agitation in individuals with dementia residing in nursing homes and to explore a variety of methodological issues in preparation for a larger study.”	- Several smaller but positive behavioral changes (less grabbing and fewer strange noises). A few changes that are contradictory (fewer resident requests for attention during the day, but more requests for attention in the evening).
	Connell et al. (31)	“A two-phase (baseline and intervention), two-group (outdoor activity program and indoor activity program) design was used to obtain preliminary data on the effect of bright light exposure during participation in a structured activity program on sleep and behavior disturbance in nursing home residents with dementia.”	- Outdoor group: Significant decline in frequency of verbal agitation. Aggression and physical agitation decline, but not significant. Indoor group no significant change.
	Detweiler et al. (26)	“To explore the effect on inappropriate behaviors of adding a wander garden to an existing dementia unit. The objective of the observational study was to assess the long-term impact of the wander garden on resident-inappropriate behaviors, incidents, and as needed medications in the effort to ultimately improve their quality of life.”	- A medium-high effect of the wander garden on CMAI scores and a reduced need for PRNs. Results of the survey of both family and staff regarding the influence of the wander garden on agitation, mood, and QoL were positive. The staff also agreed that the wander garden improved their QoL. The effect of the wander garden on incident reports was inconclusive.
	Detweiler et al. (25)	“If exposure to the wander garden decreases agitation, would there be a reduction in scheduled psychiatric medications? Second, would a reduction in PRN use, perhaps complemented by a reduction in scheduled psychotropic medications, contribute to fewer falls?”	- Increased visitation of garden appears to be related to decreased severity of falls, 30% decrease in number of falls during observation year despite dementia progression. It appears that a garden may contribute to the wellbeing of different PWD in different ways: For ambulatory residents, agitation levels were reduced [see Detweiler et al. (26)]. However, there was not much change in falls. For residents using wheelchairs, the impact of the garden on agitation levels was smaller, but there was a significant fall reduction.
	Edwards et al. (34)	“To evaluate whether a therapeutic garden can improve the quality of life of aged care residents with dementia and their carers.”	- Significant improvements in agitation of residents after creation and use of therapeutic garden.
	Liao et al. (40)	“To evaluate the effects of garden visits on mood, social interaction, cognition, and behavioral problems and to determine what type of behavioral problems and cognitive abilities among patients with dementia may be improved after visiting a garden.”	- Among the evaluated behavioral problems, staff reported that garden visits reduced residents' depression, anxiety/agitation, and aggression/anger significantly more than other behavioral problems. Staff members in the free garden use group reported that the effects of garden visits on improving residents' aggression/anger, and anxiety/agitation were significantly better than those in the unfree garden group. Garden visits had positive effects on behavioral problems, through multisensory stimulation, a feeling of independence, provoking a recall of memories, and relieving of stress.
	Mather et al. (41)	“To assess the benefit a specialized service such as the garden would give to severe Alzheimer's patients.”	- No significant differences found on disruptive behavior across three shifts and three time periods. Having access to the outdoors did not decrease aggression. Residents who showed the greatest changes over the observation period were those who used the garden the most. They showed less overall disruptive behaviors when compared to infrequent users of the garden.
	Motealleh et al. (42)	“To investigate the effect of a garden improved according to the dementia-friendly environment (DFE) characteristics on agitation, apathy, and engagement of people with dementia in one residential aged care facility.”	- No significant improvement on agitation in quantitative data. Qualitative findings indicated effectiveness of the garden in reducing agitation and restlessness of several PWD. Apathy was lower during intervention, compared to baseline.
	Murphy et al. (27)	“To reevaluate the findings of the study of Detweiler et al. (26). What is the effect of visiting the wander garden on the agitation scores of elderly dementia patients? Does the effect vary from person to person? If so, can an individual's ambulation category help explain the variability?”	- In general, a high average number of days spent in the wander garden is associated with decreased agitation scores. There is more impact for the PWD who had higher levels of agitation at the beginning than for those who had lower levels of agitation. Voluntary wander garden visits significantly lower agitation levels for ambulatory PWD; however, for the merry walker and wheelchair users there was virtually no change in CMAI scores over the course of the study. Visiting the wander garden is useful in reducing agitation level, but the rate of change depends on ambulation ability. When residents can walk without assistance, a low, medium and high usage of the garden reduces agitation: the higher the frequency the greater the decrease in agitation. For the merry walker and wheelchair users, those who had a high number of garden visits had decreased agitation levels, but medium or low garden usage was associated with unchanged or increased agitation.
	van der Velde-van Buuringen et al. (12)	“To evaluate the process (usefulness, feasibility, and applicability) of going outside daily in a nursing home garden and to explore the effect of garden use on QoL and neuropsychiatric symptoms in persons with advanced dementia.”	No significant differences were found for the participants' total and cluster scores on the NPI-NH. The intervention was observed by the caregivers to be positive for the people living with dementia through less agitated behavior.

##### 3.8.3. Other outcomes related to QoL or BPSD

Other outcomes related to QoL or BPSD included stress, sleep, and mood (see Table 1). Regarding stress, Goto et al. (36) showed that when residents observed a Japanese garden with the door open, their physiological stress was relieved, as reflected in a sustained drop in the pulse rate of the residents. The blunting of the effect when the viewing was through a glass door hints at the importance of the sense of immersion in the scene. A number of publications found positive, but also contradictory effects of garden use on sleep. For example, the study by Connell et al. (31) showed no significant change in number of wakes when comparing an outdoor and indoor group during an intervention study. By contrast, Mather et al. (41) found that residents who used the garden often showed less sleep disruption when compared to infrequent users of the garden. Lastly, most of the publications showed an overall positive effect of garden use on mood (32, 34, 35, 40, 43). White et al. (43) found a more specific time-dependent effect on mood, namely time spent outside was a non-linear predictor of change in mood score. Marked improvements in mood were associated with outdoor time of only 20 min duration and the greatest benefits were associated with an outdoor time of 80–90 min duration. After this point, the extent of positive change in mood score declined with more time spent outside.

## 4. Discussion

Overall, the results of the included studies suggested positive effects of garden use on QoL, BPSD, and other outcomes related to QoL or BPSD in people living with dementia in nursing homes. All six publications regarding QoL described positive effects of garden use on QoL in people living with dementia in nursing homes. Two thirds of the publications regarding BPSD described positive effects of garden use on BPSD in people living with dementia in nursing homes, and one third showed no significant positive, inconclusive, or contradictory effects.

Some of the publications describe an overall positive effect on QoL and BPSD, while others show a more specific effect. Perhaps there are different mechanisms that affect the influence of garden use on QoL and BPSD. For example, Hartig et al. (15) present a framework of pathways (and possibilities for effect modification by individual or contextual variables) through which the natural environment might affect the health of broad segments of the populations. The framework shows that there are direct beneficial effects of nature on stress and indirect beneficial effects, through contact with nature, on physical activity and social contacts and therefore also on health and wellbeing (15).

This systematic review found that studies examining the effect of garden use on QoL and BPSD in people living with dementia in nursing homes mostly focused on the evaluation of effects of specifically designed gardens. However, the question is whether an intervention of specifically designed nursing home gardens for people living with dementia is sufficient and adequate, or does the complex care environment of nursing homes need a more complex intervention that includes the social and organizational aspects (44–46). The theoretical framework in de Boer et al. (45) states that the literature indicates three environmental components within residential dementia care settings that impact everyday life and functioning of persons living with dementia: Physical aspects (e.g., design), social aspects (e.g., interactions with staff), and organizational aspects (e.g., attitudes that guide behavior of staff). There are barriers to garden use by people living with dementia in nursing homes, which may negatively influence the frequency of garden use, and therefore also negatively influence QoL and BPSD (20). For example, apart from the design of the garden, one of the main barriers is the perceived risk of independent use of the outdoor space, resulting in for example locked doors (20).

A limitation of this systematic review is that pooling and meta-analysis of the results of the interventions were not possible due to the use of different methods, interventions, and outcome measures in the individual studies. Overall, the majority of the studies did not describe the interventions in sufficient detail to be able to repeat the study, and very few publications provided effect sizes in the results. Whear et al. (21) discussed this problem and suggested that research in this field may benefit from an agreed set of tools to measure key outcomes, such as QoL, agitation, use of medication, or falls. However, measuring the possible effects of interventions on the daily lives of people living with dementia is difficult, because daily life is a dynamic and multidimensional concept. It involves more than just activities, for example the physical and social environments of the nursing homes (47). Future research regarding the effects of garden use on people living with dementia in nursing homes could benefit from the development of such measures that incorporate the context of the physical and social environments of the nursing homes.

Given the positive effects of garden use on QoL and BPSD in people living with dementia in nursing homes, one might expect that garden use is already incorporated in daily nursing home practice. This is, however, not the case, as demonstrated by the low numbers of people living with dementia who go outside (3). It is important to recognize that current interventions regarding garden use with a focus on changing the physical aspects in the garden environment might not be sufficient to solve this problem. Future research should focus on including all aspects (i.e., physical, social, and organizational) in the garden use intervention, for example by providing training and activities to empower staff to implement garden use in the daily life of the people living with dementia and embedding it in the culture of the nursing home. Incorporating daily garden use does not necessarily mean an additional task, but rather rearranging priorities and moving the usual activities outside part of the time (12).

### 4.1. Conclusions and implications

Garden use seems to have a positive effect on QoL and BPSD in people living with dementia in nursing homes. However, consensus regarding measurements and key outcomes, taking into account the physical, social, and organizational aspects when designing the garden use intervention, is necessary for the reliable evaluation of these interventions.

## Data availability statement

The original contributions presented in the study are included in the article/Supplementary material, further inquiries can be directed to the corresponding author.

## Author contributions

MV-v, HV, WA, and MC: study design, writing the study protocol, and writing of the manuscript. MV-v: database search. MV-v, RH-v, HV, WA, and MC: data analysis and interpretation. All authors read and approved the final version of the manuscript.
