# Supplementary material for: The effect of garden use on quality of life and behavioral and psychological symptoms of dementia in people living with dementia in nursing homes: a systematic review
Source: Front Psychiatry. 2023 Apr 12;14:1044271. doi: 10.3389/fpsyt.2023.1044271 (PMC10130442; doi:10.3389/fpsyt.2023.1044271)
Supplement: Supplementary file 1 [file Data_Sheet_1.PDF]

Supplementary Material

1 Supplementary Data: Search strategy

1.1 Review Question:

What is the effect of garden use on quality of life and behavioral and psychological symptoms of dementia in persons living with dementia in nursing homes?

1.2 Databases

1.2.1 PubMed

http://www.ncbi.nlm.nih.gov/pubmed?otool=leiden

(("Gardens"[Mesh] OR "garden"[tw] OR "gardens"[tw] OR "Gardening"[mesh] OR "gardening"[tw] OR "garden*"[tw] OR "Horticultural Therapy"[Mesh] OR "Horticultural Therapy"[tw] **OR "Horticulture"[Mesh]** OR "horticultural*"[tw] OR "outdoor space"[tw] OR "outdoor spaces"[tw] OR "outdoor area"[tw] OR "outdoor areas"[tw] OR "outdoor*"[tw] OR "Agriculture"[Mesh] OR "farms"[tw] OR "farm"[tw] OR "farming"[tw] OR agricultur*[tw] OR "External space*"[tw] OR ("Wander*"[tw] AND (**"Nature"[Mesh] OR "nature"[tw])**) OR "green house"[tw] OR "green houses"[tw] OR "outside area"[tw] OR "outside space"[tw] **OR "outside activity"[tw] OR "outside activities"[tw]**) AND ("Dementia"[Mesh] OR "dementia"[tw] OR dement*[tw] OR alzheimer*[tw] OR "primary progressive aphasia"[tw] OR "primary progressive nonfluent aphasia"[tw] OR "Creutzfeldt-Jakob"[tw] OR "CADASIL"[tw] OR "Diffuse Neurofibrillary Tangles with Calcification"[tw] OR "Frontotemporal Lobar Degeneration"[tw] OR "Pick Disease of the Brain"[tw] OR Huntington*[tw] OR "Kluver-Bucy"[tw] OR "Lewy Body"[tw] OR lewy*[tw] OR delir*[tw] OR Creutzfeldt*[tw] OR "cjd"[tw] OR Binswanger*[tw] OR Korsakoff*[tw] OR Wernicke*[tw] OR behavioral variant*[tw] OR behavioural variant*[tw] OR "bv"[tw] OR "Progressive non fluent aphasia"[tw] OR "Progressive nonfluent aphasia"[tw] OR "pnfa"[tw]) AND ("Nursing Homes"[Mesh] OR "nursing home"[tw] OR "nursing homes"[tw] OR "Intermediate Care Facilities"[tw] OR "Intermediate Care Facility"[tw] OR "Skilled Nursing Facilities"[tw] OR "Skilled Nursing Facility"[tw] OR "Homes for the Aged"[Mesh] OR "Homes for the Aged"[tw] OR "Home for the Aged"[tw] OR "Old Age Homes"[tw] OR "Old Age Home"[tw] OR "Long-Term Care"[mesh] OR "Long-Term Care"[tw] OR "Longterm Care"[tw] **OR "long term health care"[tw] OR "long term healthcare"[tw] OR "longterm health care"[tw] OR "longterm healthcare"[tw]** OR "Communal care"[tw] OR "Institution care"[tw] OR "Institutional care"[tw] OR "nursing home*"[tw] OR "care village*"[tw] OR "dementia unit*"[tw] OR "alzheimer facilit*"[tw] OR "alzheimers facilit*"[tw] OR "alzheimer's facilit*"[tw] OR "care facilit*"[tw] **OR "aged care"[tw] OR "aged health care"[tw] OR "aged healthcare"[tw] OR "care unit"[tw] OR "care units"[tw] OR "elderly care"[tw] OR "elderly health care"[tw] OR "elderly healthcare"[tw]**))

1.2.2 MEDLINE

http://gateway.ovid.com/ovidweb.cgi?T=JS&MODE=ovid&NEWS=n&PAGE=main&D=prmz

(("Gardens"/ OR "garden".mp OR "gardens".mp OR "Gardening"/ OR "gardening".mp OR garden*.mp OR "Horticultural Therapy"/ **OR exp "Horticulture"/** OR "Horticultural Therapy".mp OR horticultural*.mp OR "outdoor space".mp OR "outdoor spaces".mp OR "outdoor area".mp OR "outdoor areas".mp OR outdoor* .mp OR "park".mp OR "parks".mp OR exp "Agriculture"/ OR "farms".mp OR "farm".mp OR "farming".mp OR agricultur*.mp OR External space*.mp OR (Wander*.mp AND ("Nature"/ OR "nature".mp)) OR "green house".mp OR "green houses".mp OR "outside area".mp OR "outside space".mp OR (open* ADJ1 space*).mp OR (outdoor ADJ1 space*).mp OR (outside ADJ1 space*).mp **OR "outside activity".mp OR "outside activities".mp**) AND (exp "Dementia"/ OR "dementia".mp OR dement*.mp OR alzheimer*.mp OR "primary progressive aphasia".mp OR "primary progressive nonfluent aphasia".mp OR "Creutzfeldt-Jakob".mp OR "CADASIL".mp OR "Diffuse Neurofibrillary Tangles with Calcification".mp OR "Frontotemporal Lobar Degeneration".mp OR "Pick Disease of the Brain".mp OR Huntington*.mp OR "Kluver-Bucy".mp OR "Lewy Body".mp OR lewy*.mp OR delir*.mp OR Creutzfeldt*.mp OR cjd*.mp OR Binswanger*.mp OR Korsakoff*.mp OR Wernicke*.mp OR behavioral variant*.mp OR behavioural variant*.mp OR "bv".mp OR "Progressive non fluent aphasia".mp OR "Progressive nonfluent aphasia".mp OR "pnfa".mp OR ((cognit* OR memory) ADJ2 (impair* OR declin* OR disorder* OR disturb* OR defect* OR confus*)).mp) AND (exp "Nursing Homes"/ OR "nursing home".mp OR "nursing homes".mp OR "Intermediate Care Facilities".mp OR "Intermediate Care Facility".mp OR "Skilled Nursing Facilities".mp OR "Skilled Nursing Facility".mp OR "Homes for the Aged"/ OR "Homes for the Aged".mp OR "Home for the Aged".mp OR "Old Age Homes".mp OR "Old Age Home".mp OR exp "Long-Term Care"/ OR "Long-Term Care".mp OR "Communal care".mp OR "Institution care".mp OR "Institutional care".mp OR nursing home*.mp OR care village*.mp OR dementia unit*.mp OR alzheimer facilit*.mp OR alzheimers facilit*.mp OR alzheimer's facilit*.mp OR care facilit*.mp OR (care ADJ1 (home* OR setting* OR residence* OR unit*)).mp OR ("long term" ADJ2 care).mp OR (elderly ADJ1 care).mp OR (Residential ADJ2 (care OR unit OR home)).mp **OR "long term health care".mp OR "long term healthcare".mp OR "longterm health care".mp OR "longterm healthcare".mp OR "aged care".mp OR "aged health care".mp OR "aged healthcare".mp OR "care unit".mp OR "care units".mp OR "elderly care".mp OR "elderly health care".mp OR "elderly healthcare".mp**))

+ Adjacency-techniek niet bruikbaar in PubMed, wel in MEDLINE/Embase e.d.

1.2.3 Embase

http://ovidsp.ovid.com/ovidweb.cgi?T=JS&PAGE=main&MODE=ovid&D=oemezd

(("garden".mp OR "gardens".mp OR "Gardening"/ OR "gardening".mp OR garden*.mp OR "Horticultural Therapy"/ OR "Horticultural Therapy".mp **OR exp "Horticulture"/** OR horticultural*.mp OR "outdoor space".mp OR "outdoor spaces".mp OR "outdoor area".mp OR "outdoor areas".mp OR outdoor* .mp OR "Agriculture"/ OR "farms".mp OR "farm".mp OR "farming".mp OR agricultur*.mp OR External space*.mp OR (Wander*.mp AND "nature".mp) OR "green house".mp OR "green houses".mp OR "outside area".mp OR "outside space".mp OR (open* ADJ1 space*).mp OR (outdoor ADJ1 space*).mp OR (outside ADJ1 space*).mp **OR "outside activity".mp OR "outside activities".mp**) AND (exp *"Dementia"/ OR "dementia".ti,ab OR dement*.ti,ab OR alzheimer*.ti,ab OR "primary progressive aphasia".ti,ab OR "primary progressive nonfluent aphasia".ti,ab OR "Creutzfeldt-Jakob".ti,ab OR "CADASIL".ti,ab OR "Diffuse Neurofibrillary Tangles with Calcification".ti,ab OR "Frontotemporal Lobar Degeneration".ti,ab OR

"Pick Disease of the Brain".ti,ab OR Huntington*.ti,ab OR "Kluver-Bucy".ti,ab OR "Lewy Body".ti,ab OR lewy*.ti,ab OR delir*.ti,ab OR Creutzfeldt*.ti,ab OR cjd*.ti,ab OR Binswanger*.ti,ab OR Korsakoff*.ti,ab OR Wernicke*.ti,ab OR behavioral variant*.ti,ab OR behavioural variant*.ti,ab OR "bv".ti,ab OR "Progressive non fluent aphasia".ti,ab OR "Progressive nonfluent aphasia".ti,ab OR "pnfa".ti,ab OR ((cognit* OR memory) ADJ2 (impair* OR declin* OR disorder* OR disturb* OR defect* OR confus*)).ti,ab) AND (exp *"Nursing Home"/ OR "nursing home".ti,ab OR "nursing homes".ti,ab OR "Intermediate Care Facilities".ti,ab OR "Intermediate Care Facility".ti,ab OR "Skilled Nursing Facilities".ti,ab OR "Skilled Nursing Facility".ti,ab OR *"Home for the Aged"/ OR "Homes for the Aged".ti,ab OR "Home for the Aged".ti,ab OR "Old Age Homes".ti,ab OR "Old Age Home".ti,ab OR *"Long Term Care"/ OR "Long-Term Care".ti,ab OR "Communal care".ti,ab OR "Institution care".ti,ab OR "Institutional care".ti,ab OR nursing home*.ti,ab OR care village*.ti,ab OR dementia unit*.ti,ab OR alzheimer facilit*.ti,ab OR alzheimers facilit*.ti,ab OR alzheimer's facilit*.ti,ab OR care facilit*.ti,ab OR (care ADJ1 (home* OR setting* OR residence* OR unit*)).ti,ab OR ("long term" ADJ2 care).ti,ab OR (elderly ADJ1 care).ti,ab OR (Residential ADJ2 (care OR unit OR home)).ti,ab **OR "long term health care".ti,ab OR "long term healthcare".ti,ab OR "longterm health care".ti,ab OR "longterm healthcare".ti,ab OR "aged care".ti,ab OR "aged health care".ti,ab OR "aged healthcare".ti,ab OR "care unit".ti,ab OR "care units".ti,ab OR "elderly care".ti,ab OR "elderly health care".ti,ab OR "elderly healthcare".ti,ab**))

1.2.4 Web of Science

http://isiknowledge.com/wos

(TS=("garden" OR "gardens" OR "Gardening" OR "gardening" OR garden* OR "Horticultural Therapy" OR "Horticultural Therapy" OR horticultur* OR "outdoor space" OR "outdoor spaces" OR "outdoor area" OR "outdoor areas" OR outdoor* OR "Agriculture" OR "farms" OR "farm" OR "farming" OR agricultur* OR External space* OR (Wander* AND "nature") OR "green house" OR "green houses" OR "outside area" OR "outside space" OR (open* NEAR1 space*) OR (outdoor NEAR1 space*) OR (outside NEAR1 space*) **OR "outside activity" OR "outside activities"**) AND TS=("Dementia" OR "dementia" OR dement* OR alzheimer* OR "primary progressive aphasia" OR "primary progressive nonfluent aphasia" OR "Creutzfeldt-Jakob" OR "CADASIL" OR "Diffuse Neurofibrillary Tangles with Calcification" OR "Frontotemporal Lobar Degeneration" OR "Pick Disease of the Brain" OR Huntington* OR "Kluver-Bucy" OR "Lewy Body" OR lewy* OR delir* OR Creutzfeldt* OR cjd* OR Binswanger* OR Korsakoff* OR Wernicke* OR behavioral variant* OR behavioural variant* OR "bv" OR "Progressive non fluent aphasia" OR "Progressive nonfluent aphasia" OR "pnfa" OR ((cognit* OR memory) NEAR2 (impair* OR declin* OR disorder* OR disturb* OR defect* OR confus*))) AND TI=("Nursing Home" OR "nursing home" OR "nursing homes" OR "Intermediate Care Facilities" OR "Intermediate Care Facility" OR "Skilled Nursing Facilities" OR "Skilled Nursing Facility" OR "Home for the Aged" OR "Homes for the Aged" OR "Home for the Aged" OR "Old Age Homes" OR "Old Age Home" OR "Long Term Care" OR "Long-Term Care" OR "Communal care" OR "Institution care" OR "Institutional care" OR nursing home* OR care village* OR dementia unit* OR alzheimer facilit* OR alzheimers facilit* OR alzheimer's facilit* OR care facilit* OR (care NEAR1 (home* OR setting* OR residence* OR unit*)) OR ("long term" NEAR2 care) OR (elderly NEAR1 care) OR (Residential NEAR2 (care OR unit OR home)) **OR "aged care" OR "aged health care" OR "aged healthcare" OR "care unit" OR "care units" OR "elderly care" OR "elderly health care" OR "elderly healthcare" OR "long term health care" OR "long term healthcare" OR "longterm health care" OR "longterm healthcare"**)) OR (TS=("garden" OR "gardens" OR "Gardening" OR "gardening" OR garden* OR "Horticultural

Therapy" OR "Horticultural Therapy" OR horticultur* OR "outdoor space" OR "outdoor spaces" OR "outdoor area" OR "outdoor areas" OR outdoor* OR "Agriculture" OR "farms" OR "farm" OR "farming" OR agricultur* OR External space* OR (Wander* AND "nature") OR "green house" OR "green houses" OR "outside area" OR "outside space" OR (open* NEAR1 space*) OR (outdoor NEAR1 space*) OR (outside NEAR1 space*) **OR "outside activity" OR "outside activities"**) AND TI=("Dementia" OR "dementia" OR dement* OR alzheimer* OR "primary progressive aphasia" OR "primary progressive nonfluent aphasia" OR "Creutzfeldt-Jakob" OR "CADASIL" OR "Diffuse Neurofibrillary Tangles with Calcification" OR "Frontotemporal Lobar Degeneration" OR "Pick Disease of the Brain" OR Huntington* OR "Kluver-Bucy" OR "Lewy Body" OR lewy* OR delir* OR Creutzfeldt* OR cjd* OR Binswanger* OR Korsakoff* OR Wernicke* OR behavioral variant* OR behavioural variant* OR "bv" OR "Progressive non fluent aphasia" OR "Progressive nonfluent aphasia" OR "pnfa" OR ((cognit* OR memory) NEAR2 (impair* OR declin* OR disorder* OR disturb* OR defect* OR confus*))) AND TS=("Nursing Home" OR "nursing home" OR "nursing homes" OR "Intermediate Care Facilities" OR "Intermediate Care Facility" OR "Skilled Nursing Facilities" OR "Skilled Nursing Facility" OR "Home for the Aged" OR "Homes for the Aged" OR "Home for the Aged" OR "Old Age Homes" OR "Old Age Home" OR "Long Term Care" OR "Long-Term Care" OR "Communal care" OR "Institution care" OR "Institutional care" OR nursing home* OR care village* OR dementia unit* OR alzheimer facilit* OR alzheimers facilit* OR alzheimer's facilit* OR care facilit* OR (care NEAR1 (home* OR setting* OR residence* OR unit*)) OR ("long term" NEAR2 care) OR (elderly NEAR1 care) OR (Residential NEAR2 (care OR unit OR home)) **OR "aged care" OR "aged health care" OR "aged healthcare" OR "care unit" OR "care units" OR "elderly care" OR "elderly health care" OR "elderly healthcare" OR "long term health care" OR "long term healthcare" OR "longterm health care" OR "longterm healthcare"**)) OR (TI=("garden" OR "gardens" OR "Gardening" OR "gardening" OR garden* OR "Horticultural Therapy" OR "Horticultural Therapy" OR horticultur* OR "outdoor space" OR "outdoor spaces" OR "outdoor area" OR "outdoor areas" OR outdoor* OR "park" OR "parks" OR "Agriculture" OR "farms" OR "farm" OR "farming" OR agricultur* OR External space* OR (Wander* AND ("park" OR "parks")) OR "green house" OR "green houses" OR "outside area" OR "outside space" OR (open* NEAR1 space*) OR (outdoor NEAR1 space*) OR (outside NEAR1 space*) **OR "outside activity" OR "outside activities"**) AND TS=("Dementia" OR "dementia" OR dement* OR alzheimer* OR "primary progressive aphasia" OR "primary progressive nonfluent aphasia" OR "Creutzfeldt-Jakob" OR "CADASIL" OR "Diffuse Neurofibrillary Tangles with Calcification" OR "Frontotemporal Lobar Degeneration" OR "Pick Disease of the Brain" OR Huntington* OR "Kluver-Bucy" OR "Lewy Body" OR lewy* OR delir* OR Creutzfeldt* OR cjd* OR Binswanger* OR Korsakoff* OR Wernicke* OR behavioral variant* OR behavioural variant* OR "bv" OR "Progressive non fluent aphasia" OR "Progressive nonfluent aphasia" OR "pnfa" OR ((cognit* OR memory) NEAR2 (impair* OR declin* OR disorder* OR disturb* OR defect* OR confus*))) AND TS=("Nursing Home" OR "nursing home" OR "nursing homes" OR "Intermediate Care Facilities" OR "Intermediate Care Facility" OR "Skilled Nursing Facilities" OR "Skilled Nursing Facility" OR "Home for the Aged" OR "Homes for the Aged" OR "Home for the Aged" OR "Old Age Homes" OR "Old Age Home" OR "Long Term Care" OR "Long-Term Care" OR "Communal care" OR "Institution care" OR "Institutional care" OR nursing home* OR care village* OR dementia unit* OR alzheimer facilit* OR alzheimers facilit* OR alzheimer's facilit* OR care facilit* OR (care NEAR1 (home* OR setting* OR residence* OR unit*)) OR ("long term" NEAR2 care) OR (elderly NEAR1 care) OR (Residential NEAR2 (care OR unit OR home)) **OR "aged care" OR "aged health care" OR "aged healthcare" OR "care unit" OR "care units" OR "elderly care" OR "elderly health care" OR "elderly healthcare" OR "long term**

health care" OR "long term healthcare" OR "longterm health care" OR "longterm healthcare"))

1.2.5 Cochrane

http://www.cochranelibrary.com/

(("garden" OR "gardens" OR "Gardening" OR "gardening" OR garden* OR "Horticultural Therapy" OR "Horticultural Therapy" OR horticultur* OR "outdoor space" OR "outdoor spaces" OR "outdoor area" OR "outdoor areas" OR outdoor* OR "Agriculture" OR "farms" OR "farm" OR "farming" OR agricultur* OR External space* OR (Wander* AND "natures") OR "green house" OR "green houses" OR "outside area" OR "outside space" OR (open* AND space*) OR (outdoor AND space*) OR (outside AND space*)) AND ("Dementia" OR "dementia" OR dement* OR alzheimer* OR "primary progressive aphasia" OR "primary progressive nonfluent aphasia" OR "Creutzfeldt-Jakob" OR "CADASIL" OR "Diffuse Neurofibrillary Tangles with Calcification" OR "Frontotemporal Lobar Degeneration" OR "Pick Disease of the Brain" OR Huntington* OR "Kluver-Bucy" OR "Lewy Body" OR lewy* OR delir* OR Creutzfeldt* OR cjd* OR Binswanger* OR Korsakoff* OR Wernicke* OR behavioral variant* OR behavioural variant* OR "bv" OR "Progressive non fluent aphasia" OR "Progressive nonfluent aphasia" OR "pnfa" OR ((cognit* OR memory) AND (impair* OR declin* OR disorder* OR disturb* OR defect* OR confus*))) AND ("Nursing Home" OR "nursing home" OR "nursing homes" OR "Intermediate Care Facilities" OR "Intermediate Care Facility" OR "Skilled Nursing Facilities" OR "Skilled Nursing Facility" OR "Home for the Aged" OR "Homes for the Aged" OR "Home for the Aged" OR "Old Age Homes" OR "Old Age Home" OR "Long Term Care" OR "Long-Term Care" OR "Communal care" OR "Institution care" OR "Institutional care" OR nursing home* OR care village* OR dementia unit* OR alzheimer facilit* OR alzheimers facilit* OR alzheimer's facilit* OR care facilit* OR (care AND (home* OR setting* OR residence* OR unit*)) OR ("long term" AND care) OR (elderly AND care) OR (Residential AND (care OR unit OR home)) **OR "aged care" OR "aged health care" OR "aged healthcare" OR "care unit" OR "care units" OR "elderly care" OR "elderly health care" OR "elderly healthcare" OR "long term health care" OR "long term healthcare" OR "longterm health care" OR "longterm healthcare"**)):ti,ab,kw

1.2.6 Emcare

http://ovidsp.ovid.com/ovidweb.cgi?T=JS&NEWS=n&CSC=Y&PAGE=main&D=emcr

(("garden".mp OR "gardens".mp OR "Gardening"/ OR "gardening".mp OR garden*.mp OR "Horticultural Therapy"/ OR "Horticultural Therapy".mp **OR exp "Horticulture"/** OR horticultural*.mp OR "outdoor space".mp OR "outdoor spaces".mp OR "outdoor area".mp OR "outdoor areas".mp OR outdoor* .mp OR "Agriculture"/ OR "farms".mp OR "farm".mp OR "farming".mp OR agricultur*.mp OR External space*.mp OR (Wander*.mp AND "nature".mp) OR "green house".mp OR "green houses".mp OR "outside area".mp OR "outside space".mp OR (open* ADJ1 space*).mp OR (outdoor ADJ1 space*).mp OR (outside ADJ1 space*).mp **OR "outside activity".mp OR "outside activities".mp**) AND (exp "Dementia"/ OR "dementia".mp OR dement*.mp OR alzheimer*.mp OR "primary progressive aphasia".mp OR "primary progressive nonfluent aphasia".mp OR "Creutzfeldt-Jakob".mp OR "CADASIL".mp OR "Diffuse Neurofibrillary Tangles with Calcification".mp OR "Frontotemporal Lobar Degeneration".mp OR "Pick Disease of the Brain".mp OR Huntington*.mp OR "Kluver-Bucy".mp OR "Lewy Body".mp OR lewy*.mp OR delir*.mp OR Creutzfeldt*.mp OR cjd*.mp OR Binswanger*.mp OR Korsakoff*.mp OR

Wernicke*.mp OR behavioral variant*.mp OR behavioural variant*.mp OR "bv".mp OR "Progressive non fluent aphasia".mp OR "Progressive nonfluent aphasia".mp OR "pnfa".mp OR ((cognit* OR memory) ADJ2 (impair* OR declin* OR disorder* OR disturb* OR defect* OR confus*)).mp) AND (exp "Nursing Home"/ OR "nursing home".mp OR "nursing homes".mp OR "Intermediate Care Facilities".mp OR "Intermediate Care Facility".mp OR "Skilled Nursing Facilities".mp OR "Skilled Nursing Facility".mp OR "Home for the Aged"/ OR "Homes for the Aged".mp OR "Home for the Aged".mp OR "Old Age Homes".mp OR "Old Age Home".mp OR "Long Term Care"/ OR "Long-Term Care".mp OR "Communal care".mp OR "Institution care".mp OR "Institutional care".mp OR nursing home*.mp OR care village*.mp OR dementia unit*.mp OR alzheimer facilit*.mp OR alzheimers facilit*.mp OR alzheimer's facilit*.mp OR care facilit*.mp OR (care ADJ1 (home* OR setting* OR residence* OR unit*)).mp OR ("long term" ADJ2 care).mp OR (elderly ADJ1 care).mp OR (Residential ADJ2 (care OR unit OR home)).mp **OR "long term health care".mp OR "long term healthcare".mp OR "longterm health care".mp OR "longterm healthcare".mp OR "aged care".mp OR "aged health care".mp OR "aged healthcare".mp OR "care unit".mp OR "care units".mp OR "elderly care".mp OR "elderly health care".mp OR "elderly healthcare".mp**))

1.2.7 PsycINFO

http://search.ebscohost.com/login.aspx?authtype=ip,uid&profile=lumc&defaultdb=psyh

TI(("garden" OR "gardens" OR "Gardening" OR "gardening" OR garden* OR "Horticultural Therapy" OR "Horticultural Therapy" OR horticultur* OR "outdoor space" OR "outdoor spaces" OR "outdoor area" OR "outdoor areas" OR outdoor* OR "Agriculture" OR "farms" OR "farm" OR "farming" OR agricultur* OR External space* OR (Wander* AND "nature") OR "green house" OR "green houses" OR "outside area" OR "outside space" OR (open* NEAR1 space*) OR (outdoor NEAR1 space*) OR (outside NEAR1 space*) **OR "outside activity" OR "outside activities"**) AND ("Dementia" OR "dementia" OR dement* OR alzheimer* OR "primary progressive aphasia" OR "primary progressive nonfluent aphasia" OR "Creutzfeldt-Jakob" OR "CADASIL" OR "Diffuse Neurofibrillary Tangles with Calcification" OR "Frontotemporal Lobar Degeneration" OR "Pick Disease of the Brain" OR Huntington* OR "Kluver-Bucy" OR "Lewy Body" OR lewy* OR delir* OR Creutzfeldt* OR cjd* OR Binswanger* OR Korsakoff* OR Wernicke* OR behavioral variant* OR behavioural variant* OR "bv" OR "Progressive non fluent aphasia" OR "Progressive nonfluent aphasia" OR "pnfa" OR ((cognit* OR memory) NEAR2 (impair* OR declin* OR disorder* OR disturb* OR defect* OR confus*))) AND ("Nursing Home" OR "nursing home" OR "nursing homes" OR "Intermediate Care Facilities" OR "Intermediate Care Facility" OR "Skilled Nursing Facilities" OR "Skilled Nursing Facility" OR "Home for the Aged" OR "Homes for the Aged" OR "Home for the Aged" OR "Old Age Homes" OR "Old Age Home" OR "Long Term Care" OR "Long-Term Care" OR "Communal care" OR "Institution care" OR "Institutional care" OR nursing home* OR care village* OR dementia unit* OR alzheimer facilit* OR alzheimers facilit* OR alzheimer's facilit* OR care facilit* OR (care NEAR1 (home* OR setting* OR residence* OR unit*)) OR ("long term" NEAR2 care) OR (elderly NEAR1 care) OR (Residential NEAR2 (care OR unit OR home)) **OR "long term health care" OR "long term healthcare" OR "longterm health care" OR "longterm healthcare" OR "aged care" OR "aged health care" OR "aged healthcare" OR "care unit" OR "care units" OR "elderly care" OR "elderly health care" OR "elderly healthcare"**)) OR SU(("garden" OR "gardens" OR "Gardening" OR "gardening" OR garden* OR "Horticultural Therapy" OR "Horticultural Therapy" OR horticultur* OR "outdoor space" OR "outdoor spaces" OR "outdoor area" OR "outdoor areas" OR outdoor* OR "Agriculture" OR "farms" OR "farm" OR "farming" OR agricultur* OR External space* OR (Wander* AND "nature")

OR "green house" OR "green houses" OR "outside area" OR "outside space" OR (open* NEAR1 space*) OR (outdoor NEAR1 space*) OR (outside NEAR1 space*) **OR "outside activity" OR "outside activities"**) AND ("Dementia" OR "dementia" OR dement* OR alzheimer* OR "primary progressive aphasia" OR "primary progressive nonfluent aphasia" OR "Creutzfeldt-Jakob" OR "CADASIL" OR "Diffuse Neurofibrillary Tangles with Calcification" OR "Frontotemporal Lobar Degeneration" OR "Pick Disease of the Brain" OR Huntington* OR "Kluver-Bucy" OR "Lewy Body" OR lewy* OR delir* OR Creutzfeldt* OR cjd* OR Binswanger* OR Korsakoff* OR Wernicke* OR behavioral variant* OR behavioural variant* OR "bv" OR "Progressive non fluent aphasia" OR "Progressive nonfluent aphasia" OR "pnfa" OR ((cognit* OR memory) NEAR2 (impair* OR declin* OR disorder* OR disturb* OR defect* OR confus*))) AND ("Nursing Home" OR "nursing home" OR "nursing homes" OR "Intermediate Care Facilities" OR "Intermediate Care Facility" OR "Skilled Nursing Facilities" OR "Skilled Nursing Facility" OR "Home for the Aged" OR "Homes for the Aged" OR "Home for the Aged" OR "Old Age Homes" OR "Old Age Home" OR "Long Term Care" OR "Long-Term Care" OR "Communal care" OR "Institution care" OR "Institutional care" OR nursing home* OR care village* OR dementia unit* OR alzheimer facilit* OR alzheimers facilit* OR alzheimer's facilit* OR care facilit* OR (care NEAR1 (home* OR setting* OR residence* OR unit*)) OR ("long term" NEAR2 care) OR (elderly NEAR1 care) OR (Residential NEAR2 (care OR unit OR home)) **OR "long term health care" OR "long term healthcare" OR "longterm health care" OR "longterm healthcare" OR "aged care" OR "aged health care" OR "aged healthcare" OR "care unit" OR "care units" OR "elderly care" OR "elderly health care" OR "elderly healthcare"**)) OR MA(("garden" OR "gardens" OR "Gardening" OR "gardening" OR garden* OR "Horticultural Therapy" OR "Horticultural Therapy" OR horticultur* OR "outdoor space" OR "outdoor spaces" OR "outdoor area" OR "outdoor areas" OR outdoor* OR "Agriculture" OR "farms" OR "farm" OR "farming" OR agricultur* OR External space* OR (Wander* AND "nature") OR "green house" OR "green houses" OR "outside area" OR "outside space" OR (open* NEAR1 space*) OR (outdoor NEAR1 space*) OR (outside NEAR1 space*) **OR "outside activity" OR "outside activities"**) AND ("Dementia" OR "dementia" OR dement* OR alzheimer* OR "primary progressive aphasia" OR "primary progressive nonfluent aphasia" OR "Creutzfeldt-Jakob" OR "CADASIL" OR "Diffuse Neurofibrillary Tangles with Calcification" OR "Frontotemporal Lobar Degeneration" OR "Pick Disease of the Brain" OR Huntington* OR "Kluver-Bucy" OR "Lewy Body" OR lewy* OR delir* OR Creutzfeldt* OR cjd* OR Binswanger* OR Korsakoff* OR Wernicke* OR behavioral variant* OR behavioural variant* OR "bv" OR "Progressive non fluent aphasia" OR "Progressive nonfluent aphasia" OR "pnfa" OR ((cognit* OR memory) NEAR2 (impair* OR declin* OR disorder* OR disturb* OR defect* OR confus*))) AND ("Nursing Home" OR "nursing home" OR "nursing homes" OR "Intermediate Care Facilities" OR "Intermediate Care Facility" OR "Skilled Nursing Facilities" OR "Skilled Nursing Facility" OR "Home for the Aged" OR "Homes for the Aged" OR "Home for the Aged" OR "Old Age Homes" OR "Old Age Home" OR "Long Term Care" OR "Long-Term Care" OR "Communal care" OR "Institution care" OR "Institutional care" OR nursing home* OR care village* OR dementia unit* OR alzheimer facilit* OR alzheimers facilit* OR alzheimer's facilit* OR care facilit* OR (care NEAR1 (home* OR setting* OR residence* OR unit*)) OR ("long term" NEAR2 care) OR (elderly NEAR1 care) OR (Residential NEAR2 (care OR unit OR home)) **OR "long term health care" OR "long term healthcare" OR "longterm health care" OR "longterm healthcare" OR "aged care" OR "aged health care" OR "aged healthcare" OR "care unit" OR "care units" OR "elderly care" OR "elderly health care" OR "elderly healthcare"**))

1.2.8 Academic Search Premier

https://databases.library.leiden.edu/redirect/9900246985903027 11

TI(("garden" OR "gardens" OR "Gardening" OR "gardening" OR garden* OR "Horticultural Therapy" OR "Horticultural Therapy" OR horticultur* OR "outdoor space" OR "outdoor spaces" OR "outdoor area" OR "outdoor areas" OR outdoor* OR "Agriculture" OR "farms" OR "farm" OR "farming" OR agricultur* OR External space* OR (Wander* AND "nature") OR "green house" OR "green houses" OR "outside area" OR "outside space" OR (open* NEAR1 space*) OR (outdoor NEAR1 space*) OR (outside NEAR1 space*) **OR "outside activity" OR "outside activities"**) AND ("Dementia" OR "dementia" OR dement* OR alzheimer* OR "primary progressive aphasia" OR "primary progressive nonfluent aphasia" OR "Creutzfeldt-Jakob" OR "CADASIL" OR "Diffuse Neurofibrillary Tangles with Calcification" OR "Frontotemporal Lobar Degeneration" OR "Pick Disease of the Brain" OR Huntington* OR "Kluver-Bucy" OR "Lewy Body" OR lewy* OR delir* OR Creutzfeldt* OR cjd* OR Binswanger* OR Korsakoff* OR Wernicke* OR behavioral variant* OR behavioural variant* OR "bv" OR "Progressive non fluent aphasia" OR "Progressive nonfluent aphasia" OR "pnfa" OR ((cognit* OR memory) NEAR2 (impair* OR declin* OR disorder* OR disturb* OR defect* OR confus*))) AND ("Nursing Home" OR "nursing home" OR "nursing homes" OR "Intermediate Care Facilities" OR "Intermediate Care Facility" OR "Skilled Nursing Facilities" OR "Skilled Nursing Facility" OR "Home for the Aged" OR "Homes for the Aged" OR "Home for the Aged" OR "Old Age Homes" OR "Old Age Home" OR "Long Term Care" OR "Long-Term Care" OR "Communal care" OR "Institution care" OR "Institutional care" OR nursing home* OR care village* OR dementia unit* OR alzheimer facilit* OR alzheimers facilit* OR alzheimer's facilit* OR care facilit* OR (care NEAR1 (home* OR setting* OR residence* OR unit*)) OR ("long term" NEAR2 care) OR (elderly NEAR1 care) OR (Residential NEAR2 (care OR unit OR home)) **OR "long term health care" OR "long term healthcare" OR "longterm health care" OR "longterm healthcare" OR "aged care" OR "aged health care" OR "aged healthcare" OR "care unit" OR "care units" OR "elderly care" OR "elderly health care" OR "elderly healthcare"**)) OR SU(("garden" OR "gardens" OR "Gardening" OR "gardening" OR garden* OR "Horticultural Therapy" OR "Horticultural Therapy" OR horticultur* OR "outdoor space" OR "outdoor spaces" OR "outdoor area" OR "outdoor areas" OR outdoor* OR "Agriculture" OR "farms" OR "farm" OR "farming" OR agricultur* OR External space* OR (Wander* AND "nature") OR "green house" OR "green houses" OR "outside area" OR "outside space" OR (open* NEAR1 space*) OR (outdoor NEAR1 space*) OR (outside NEAR1 space*) **OR "outside activity" OR "outside activities"**) AND ("Dementia" OR "dementia" OR dement* OR alzheimer* OR "primary progressive aphasia" OR "primary progressive nonfluent aphasia" OR "Creutzfeldt-Jakob" OR "CADASIL" OR "Diffuse Neurofibrillary Tangles with Calcification" OR "Frontotemporal Lobar Degeneration" OR "Pick Disease of the Brain" OR Huntington* OR "Kluver-Bucy" OR "Lewy Body" OR lewy* OR delir* OR Creutzfeldt* OR cjd* OR Binswanger* OR Korsakoff* OR Wernicke* OR behavioral variant* OR behavioural variant* OR "bv" OR "Progressive non fluent aphasia" OR "Progressive nonfluent aphasia" OR "pnfa" OR ((cognit* OR memory) NEAR2 (impair* OR declin* OR disorder* OR disturb* OR defect* OR confus*))) AND ("Nursing Home" OR "nursing home" OR "nursing homes" OR "Intermediate Care Facilities" OR "Intermediate Care Facility" OR "Skilled Nursing Facilities" OR "Skilled Nursing Facility" OR "Home for the Aged" OR "Homes for the Aged" OR "Home for the Aged" OR "Old Age Homes" OR "Old Age Home" OR "Long Term Care" OR "Long-Term Care" OR "Communal care" OR "Institution care" OR "Institutional care" OR nursing home* OR care village* OR dementia unit* OR alzheimer facilit* OR alzheimers facilit* OR alzheimer's facilit* OR care facilit* OR (care NEAR1 (home* OR setting* OR residence* OR unit*)) OR ("long term" NEAR2 care) OR (elderly NEAR1 care) OR (Residential NEAR2 (care OR unit OR home)) **OR "long term health care" OR "long term healthcare" OR "longterm health care" OR "longterm healthcare" OR "aged care" OR "aged health care" OR "aged healthcare" OR "care unit" OR "care units" OR "elderly care" OR "elderly health care" OR "elderly healthcare"**))

OR KW(("garden" OR "gardens" OR "Gardening" OR "gardening" OR garden* OR "Horticultural Therapy" OR "Horticultural Therapy" OR horticultur* OR "outdoor space" OR "outdoor spaces" OR "outdoor area" OR "outdoor areas" OR outdoor* OR "Agriculture" OR "farms" OR "farm" OR "farming" OR agricultur* OR External space* OR (Wander* AND "nature") OR "green house" OR "green houses" OR "outside area" OR "outside space" OR (open* NEAR1 space*) OR (outdoor NEAR1 space*) OR (outside NEAR1 space*) **OR "outside activity" OR "outside activities"**) AND ("Dementia" OR "dementia" OR dement* OR alzheimer* OR "primary progressive aphasia" OR "primary progressive nonfluent aphasia" OR "Creutzfeldt-Jakob" OR "CADASIL" OR "Diffuse Neurofibrillary Tangles with Calcification" OR "Frontotemporal Lobar Degeneration" OR "Pick Disease of the Brain" OR Huntington* OR "Kluver-Bucy" OR "Lewy Body" OR lewy* OR delir* OR Creutzfeldt* OR cjd* OR Binswanger* OR Korsakoff* OR Wernicke* OR behavioral variant* OR behavioural variant* OR "bv" OR "Progressive non fluent aphasia" OR "Progressive nonfluent aphasia" OR "pnfa" OR ((cognit* OR memory) NEAR2 (impair* OR declin* OR disorder* OR disturb* OR defect* OR confus*))) AND ("Nursing Home" OR "nursing home" OR "nursing homes" OR "Intermediate Care Facilities" OR "Intermediate Care Facility" OR "Skilled Nursing Facilities" OR "Skilled Nursing Facility" OR "Home for the Aged" OR "Homes for the Aged" OR "Home for the Aged" OR "Old Age Homes" OR "Old Age Home" OR "Long Term Care" OR "Long-Term Care" OR "Communal care" OR "Institution care" OR "Institutional care" OR nursing home* OR care village* OR dementia unit* OR alzheimer facilit* OR alzheimers facilit* OR alzheimer's facilit* OR care facilit* OR (care NEAR1 (home* OR setting* OR residence* OR unit*)) OR ("long term" NEAR2 care) OR (elderly NEAR1 care) OR (Residential NEAR2 (care OR unit OR home)) **OR "long term health care" OR "long term healthcare" OR "longterm health care" OR "longterm healthcare" OR "aged care" OR "aged health care" OR "aged healthcare" OR "care unit" OR "care units" OR "elderly care" OR "elderly health care" OR "elderly healthcare"**))

1.2.9 Social Services Abstracts / Sociological Abstracts

https://search.proquest.com/socialservices?accountid=12045

http://databases.library.leiden.edu/?bibid=9900273330303027 11&redirect=true

NOFT

(("garden" OR "gardens" OR "Gardening" OR "gardening" OR garden* OR "Horticultural Therapy" OR "Horticultural Therapy" OR horticultur* OR "outdoor space" OR "outdoor spaces" OR "outdoor area" OR "outdoor areas" OR outdoor* OR "Agriculture" OR "farms" OR "farm" OR "farming" OR agricultur* OR External space* OR (Wander* AND "nature") OR "green house" OR "green houses" OR "outside area" OR "outside space" OR (open* NEAR1 space*) OR (outdoor NEAR1 space*) OR (outside NEAR1 space*) **OR "outside activity" OR "outside activities"**) AND ("Dementia" OR "dementia" OR dement* OR alzheimer* OR "primary progressive aphasia" OR "primary progressive nonfluent aphasia" OR "Creutzfeldt-Jakob" OR "CADASIL" OR "Diffuse Neurofibrillary Tangles with Calcification" OR "Frontotemporal Lobar Degeneration" OR "Pick Disease of the Brain" OR Huntington* OR "Kluver-Bucy" OR "Lewy Body" OR lewy* OR delir* OR Creutzfeldt* OR cjd* OR Binswanger* OR Korsakoff* OR Wernicke* OR behavioral variant* OR behavioural variant* OR "bv" OR "Progressive non fluent aphasia" OR "Progressive nonfluent aphasia" OR "pnfa" OR ((cognit* OR memory) NEAR2 (impair* OR declin* OR disorder* OR disturb* OR defect* OR confus*))) AND ("Nursing Home" OR "nursing home" OR "nursing homes" OR "Intermediate Care Facilities" OR "Intermediate Care Facility" OR "Skilled Nursing Facilities" OR "Skilled Nursing Facility" OR "Home

for the Aged" OR "Homes for the Aged" OR "Home for the Aged" OR "Old Age Homes" OR "Old Age Home" OR "Long Term Care" OR "Long-Term Care" OR "Communal care" OR "Institution care" OR "Institutional care" OR nursing home* OR care village* OR dementia unit* OR alzheimer facilit* OR alzheimers facilit* OR alzheimer's facilit* OR care facilit* OR (care NEAR1 (home* OR setting* OR residence* OR unit*)) OR ("long term" NEAR2 care) OR (elderly NEAR1 care) OR (Residential NEAR2 (care OR unit OR home)) **OR "long term health care" OR "long term healthcare" OR "longterm health care" OR "longterm healthcare" OR "aged care" OR "aged health care" OR "aged healthcare" OR "care unit" OR "care units" OR "elderly care" OR "elderly health care" OR "elderly healthcare"**))
